# The bZIP transcription factor TabZIP15 improves salt stress tolerance in wheat

**DOI:** 10.1111/pbi.13453

**Published:** 2020-08-13

**Authors:** Chenxi Bi, Yuehua Yu, Chunhao Dong, Yuxin Yang, Yiqian Zhai, Fengping Du, Chuan Xia, Zhiyong Ni, Xiuying Kong, Lichao Zhang

**Affiliations:** ^1^ Key Laboratory for Crop Gene Resources and Germplasm Enhancement MOA National Key Facility for Crop Gene Resources and Genetic Improvement Institute of Crop Sciences Chinese Academy of Agricultural Sciences Beijing China; ^2^ College of Agronomy Xinjiang Agricultural University Urumqi China

**Keywords:** wheat, bZIP, transcriptional factor, salt stress tolerance

As one of the most important staple foods, wheat plays a crucial role in sustaining food security. However, its production is greatly threatened by abiotic stresses such as salt (Khataar *et al*., 2018; Qi *et al*., 2019). Therefore, discovering the genes involved in abiotic stress tolerance and cultivating genetically modified plant varieties with enhanced stress tolerance are currently among the most important goals for plant breeders (Genc *et al*., 2019). To identify wheat transcriptional factor (TF) genes that are essential for regulating abiotic stress tolerance, 1455 wheat TF‐encoding genes from full‐length cDNA sequence libraries were tested for their expression under abiotic stress treatment conditions. One gene, *TabZIP15* (*TraesCS7A02G488600*), was identified as a salt stress‐responsive gene in wheat.

*TabZIP15* contains six exons and five introns in its structure (Figure 1a) and encodes a bZIP TF (Figure 1b). In the roots at the seedling stage, *TabZIP15* expression was induced quickly and peaked within 1 h following salt stress treatment, and the expression level was approximately 2.4‐fold greater than that before treatment (Figure 1c). TabZIP15 is a nuclear‐localized protein (Figure 1d) with transcriptional activation activity in yeast (Figure 1e). Moreover, we also found that TabZIP15 has an affinity for the ABA‐responsive element (ABRE) *Cis*‐element using the yeast one‐hybrid assay system (Figure 1f).

**Figure 1:**
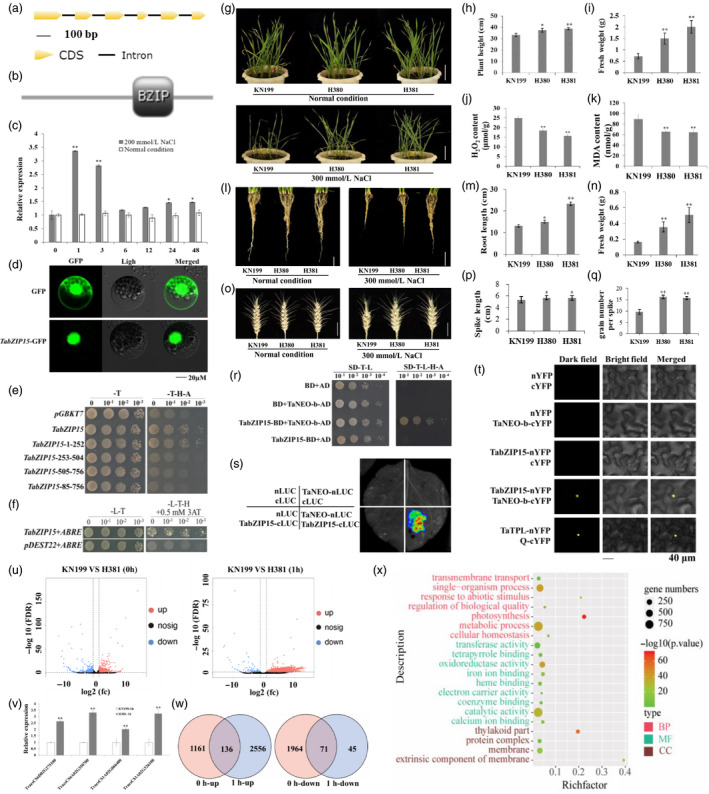
Functional characterization of *TabZIP15* in the regulation of salt stress in wheat. (a and b) Schematic of the *TabZIP15* gene structure (a) and its putative coding protein (b). (c) Expression patterns of the *TabZIP15* gene in roots at different time points after salt stress treatment at the seedling stage. (d) Subcellular localization of the TabZIP15 protein in wheat leaf protoplasts. (e) Test of the transcriptional activity of the full‐length and truncated fragments of TabZIP15. (f) TabZIP15 binds specifically to the ABRE *Cis*‐element in yeast cells. (g) Phenotypes of KN199 and *TabZIP15* transgenic plants under normal and salt stress conditions. Bars = 10 cm. (h‐k) The plant height (h), aboveground fresh weight (i) and malondialdehyde (MDA) (j) and H_2_O_2_ contents (k) of seedlings under the salt stress condition. (l) Root phenotypes of KN199 plants and *TabZIP15* transgenic wheat plants under normal and salt stress conditions. Bars = 5 cm. (m and n) The length (m) and fresh weight (n) of roots under the salt stress condition. (o) Spike phenotypes of KN199 and *TabZIP15* transgenic plants in the reproductive stage under normal and salt stress conditions. Bars = 2 cm. (p and q) The spike length (p) and grain number per spike (q) of KN199 and *TabZIP15* transgenic plants under the salt stress condition. (r) Yeast two‐hybrid assay shows that TabZIP15 interacts with TaENO‐b. (s and t) Firefly luciferase complementation imaging (s) and bimolecular fluorescence complementation (t) assays confirming the interaction between TabZIP15 and TaNEO‐b. Two known interacting proteins, TaTPL and Q, were used as positive controls. (u) Differentially expressed genes (DEGs) between KN199 and H381 plants at 0 h and 1 h after salt stress treatment, as shown by volcano plots. (v) Four genes were selected to verify the transcriptome results. (w) Venn diagrams demonstrating the comparison of the up‐regulated and down‐regulated DEGs between 0 h and 1 h after salt stress treatment. (x) Gene Ontology enrichment analyses of the DEGs whose expression was up‐regulated specifically at 1 h after salt stress treatment in H381 plants. BP: biological process, MF: molecular function, CC: cellular component. [Colour figure can be viewed at wileyonlinelibrary.com]

To determine the role of the *TabZIP15* gene in the regulation of salt stress tolerance in wheat, transgenic lines of the wheat variety Kenong 199 (KN199) overexpressing *TabZIP15* under the control of the maize Ubi promoter were generated. Under normal conditions, no obvious phenotypic variation was detected between KN199 plants and the transgenic lines. However, after 20 days of salt stress treatment with 300 mm NaCl, although both the KN199 and transgenic plants displayed a certain degree of wilting and inhibited growth, the wilting degree was obviously lower in the transgenic plants than in the KN199 plants (Figure 1g). In addition, the transgenic plants presented obvious increases in height and aboveground fresh weight, and significant decreases in malondialdehyde (MDA) and H_2_O_2_ contents (Figure 1h‐k). Considering that the root system is closely related to plant abiotic stress tolerance, we also examined the morphology of the roots and found no obvious differences in root morphology, length or fresh weight between the KN199 plants and transgenic plants under normal growth conditions. However, under salt stress conditions, although the root growth of both the transgenic plants and KN199 plants was inhibited, the transgenic plants had a larger root system than the KN199 plants (Figure 1l), and the root length and fresh weight were greater in the transgenic lines than in the KN199 plants (Figure 1m and n). In the reproductive stage, the spikes of the transgenic plants were almost the same as those of the KN199 plants under normal growth conditions, while the spike length was longer and the number of grains per spike was significantly higher in the transgenic plants than in the KN199 plants (Figure 1o‐q). Together, these results suggest that the *TabZIP15* gene is involved in the regulation of wheat salt stress tolerance.

To better understand how TabZIP15 is involved in the wheat stress response, the truncated TabZIP15 was used to screen the yeast library. The results showed that the enolase protein TaENO‐b (KC342470.1) could physically interact with TabZIP15 (Figure 1r). We then conducted luciferase complementation imaging and bimolecular fluorescence complementation assays in *Nicotiana benthamiana* and confirmed the interaction between TabZIP15 and TaENO‐b (Figure 1s and t). TaENO‐b is an enolase protein that catalyses the reversible dehydration of 2‐phospho‐D‐glycerate to phosphoenolpyruvate as part of the glycolytic and gluconeogenesis pathways. Together, these results indicated that TabZIP15 may interact with enolase TaENO‐b to participate in the regulation of the glycolysis and gluconeogenesis pathways, thus improving salt stress tolerance in wheat.

To further investigate the putative mechanisms of the *TabZIP15* gene, we used RNA sequencing (RNA‐seq) transcriptomic analysis to identify differentially expressed genes (DEGs) (|log2 fold change [FC]| > 1, false discovery rate [FDR] < 0.05) between the KN199 and H381 transgenic plants before (0 h) and after salt stress treatment (1 h) at the seedling stage (Audic and Claverie, 1997; Mariani *et al*., 2003). As a result, a total of 3332 DEGs were found between KN199 and H381 plants at 0 h, including 1297 up‐regulated and 2035 down‐regulated DEGs in H381. At 1 h, 2807 DEGs were identified between KN199 and H381 plants, including 2691 up‐regulated and 116 down‐regulated DEGs in H381 plants (Figure 1u). We then tested the expression of four genes by qRT‐PCR, and the results were consistent with the transcriptome analysis results (Figure 1v). Considering that the genes whose expression was specifically induced or inhibited after salt stress treatment may have more important roles in regulating salt tolerance in the transgenic plants, we further compared the DEGs between 0 h and 1 h. The results showed that the expression of 2556 DEGs was specifically up‐regulated after salt treatment, while the expression of 45 DEGs was specifically down‐regulated (Figure 1w). The large difference in the numbers of up‐regulated and down‐regulated genes may reflect TabZIP15’s role as a transcriptional activator. Gene Ontology (GO) enrichment analysis was performed for the DEGs that were specifically up‐regulated at 1 h in transgenic plants. The 2556 genes were mainly enriched in 20 biological processes, including metabolic processes (GO:0008152), iron ion binding (GO:0005506) and the response to abiotic stimuli (GO:0009628; Figure 1x). Among these GO terms, the metabolic processes item enriched more than 900 DEGs, suggesting that the genes involved in metabolic regulation may play important roles in improving the salt tolerance of wheat through *TabZIP15*. Furthermore, more than 30 DEGs were enriched in the response to abiotic stimulus GO term. These DEGs may also regulate salt stress tolerance in wheat. In the future, we will analyse these DEGs in depth, which will help us to further understand the mechanism of the *TabZIP15* gene regulating salt tolerance in wheat.

Discovering stress‐responsive genes and studying their roles via genetic modification approaches is necessary for the development of plants with enhanced stress tolerance (Abhinandan *et al*., 2018; Hasegawa *et al*., 2000; Zhu, 2016). In this study, we characterized a salt‐induced bZIP gene, *TabZIP15*, in wheat. Based on the performance of *TabZIP15* in transgenic plants, we propose that manipulating the expression of this gene leads to enhanced salt stress tolerance. These results improve our understanding of the roles of wheat bZIP protein in the plant response to abiotic stress and highlight a candidate gene for wheat improvement.

## Conflict of interest

The authors declare no conflict of interest.

## Author contributions

X.K. and L.Z. conceived the study and designed the experiments. C.B., C.D., Y.Y., C.J., Y.Y., Y.Z., C.X. and F.D. performed the experiments. L.Z., X.K., Z.N., C.B., Y.Y. and Y.Y. wrote the manuscript.
